# Preparation and Characterization of Polyphenylsulfone (PPSU) Membranes for Biogas Upgrading

**DOI:** 10.3390/ma13122847

**Published:** 2020-06-25

**Authors:** Wojciech Kujawski, Guoqiang Li, Bart Van der Bruggen, Nerijus Pedišius, Jurij Tonkonogij, Andrius Tonkonogovas, Arūnas Stankevičius, Justas Šereika, Nora Jullok, Joanna Kujawa

**Affiliations:** 1 Faculty of Chemistry, Nicolaus Copernicus University in Toruń, 7 Gagarina Street, 87-100 Toruń, Poland; grantli@doktorant.umk.pl; 2 Leuven KU, Department of Chemical Engineering, Process Engineering for Sustainable Systems, W. de Croylaan 46, BE-3001 Heverlee, Belgium; bart.vanderbruggen@kuleuven.be (B.V.d.B.); marie9581@yahoo.com (N.J.); 3 Laboratory of Heat-Equipment Research and Testing, Lithuanian Energy Institute, 3 Breslaujos Street, 44403 Kaunas, Lithuania; Nerijus.Pedisius@lei.lt (N.P.); Jurij.Tonkonogij@gmail.com (J.T.); Andrius.Tonkonogovas@lei.lt (A.T.); Arunas.Stankevicius@lei.lt (A.S.); Justas.Sereika@lei.lt (J.Š.); 4 Centre of Excellence for Biomass Utilization, School of Bioprocess Engineering, Universiti Malaysia Perlis, Kompleks Pusat Pengajian Jejawi 3, Jejawi 02600, Perlis, Malaysia

**Keywords:** polyphenylsulfone (PPSU) membranes, gas separation, glycerin, silica nanoparticles, biogas upgrading

## Abstract

Asymmetric polyphenylsulfone (PPSU) membranes were fabricated by a non-solvent induced phase inversion method. Glycerin and silica nanoparticles were added into the polymer solution to investigate their effects on the material properties and gas separation performance of prepared membranes. The morphology and structure of PPSU membranes were analyzed by scanning electron microscopy (SEM), the surface roughness of the selective layer was analyzed by atomic force microscopy (AFM), and the surface free energy was calculated based on the contact angle measurements by using various solvents. The gas separation performance of PPSU membranes was estimated by measuring the permeability of CO_2_ and CH_4_. The addition of glycerin as a nonsolvent into the polymer solution changed the cross-section structure from finger-like structure into sponge-like structure due to the delayed liquid-liquid demixing process, which was confirmed by SEM analysis. The incorporation of silica nanoparticles into PPSU membranes slightly increased the hydrophilicity, which was confirmed by water contact angle results. PPSU membrane fabricated from the polymer solution containing 10 wt.% glycerin showed the best CO_2_/CH_4_ selectivity of 3.86 and the CO_2_ permeability of 1044.01 Barrer. Mixed matrix PPSU membrane containing 0.1 wt.% silica nanoparticles showed the CO_2_/CH_4_ selectivity of 3.16 and the CO_2_ permeability of 1202.77 Barrer.

## 1. Introduction

**Figure 1:**
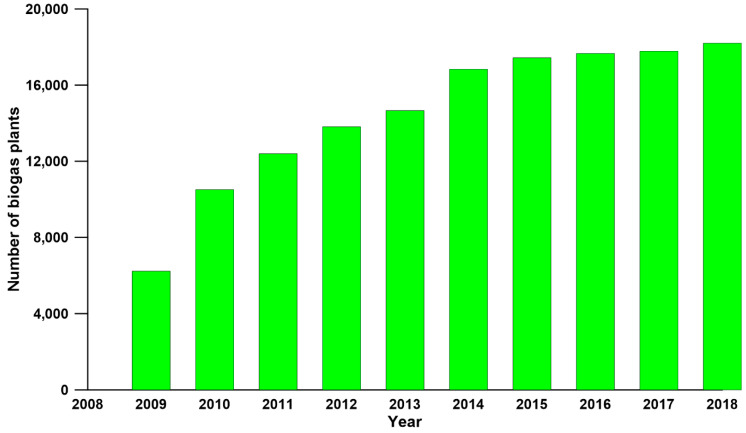
Number of biogas plants in Europe from 2009 to 2018.

Biogas has gained significant interest due to its ability to mitigate carbon dioxide emissions and pollution issues. As a renewable source of energy, it is a promising alternative to conventional fossil fuels [1,2]. According to the European Biogas Association data (Figure 1), the number of biogas plants in Europe has been increasing since 2009 and reached 18,202 in 2018 [3,4]. Biogas generally generates from biomass anaerobic digestion and mainly consists of methane (55–65%), carbon dioxide (30–45%) and other minor parts, such as water vapor and hydrogen sulfide. The composition of biogas differs due to different biomass sources [5,6,7]. The concentration of carbon dioxide should be decreased down to 2% to meet the pipeline specification. Moreover, lowering the concentration of carbon dioxide increases the heat value and combustion efficiency of biogas [6,8]. Therefore, upgrading the raw biogas by the removal of carbon dioxide is extremely important to produce biomethane for practical utilization.

Traditional gas separation technologies such as physical-chemical absorption, solid adsorption, and cryogenic distillation have been widely used in upgrading biogas [9,10,11,12]. However, these technologies are not optimal for the removal of carbon dioxide from biogas because of their high energy consumption and process complexity. Considering these drawbacks of the conventional gas separation technologies, membrane-based gas separation attracted wide attention due to its high energy efficiency, smaller footprint, low capital cost, reliability, and operational simplicity [1,13,14].

Different types of membranes have been applied in CO_2_/CH_4_ separation. Polymeric membranes show the trade-off relationship between separation factor and gas permeability, limiting their further application in gas separation process [15]. Recently, mixed matrix membranes have been investigated intensively to improve the gas permeability and gas separation factor simultaneously [13,14,16,17,18,19,20,21]. Moreover, the facilitated transport membranes have been prepared by incorporating gas carriers into the membranes to improve the gas separation performance [22,23,24].

The successful implementation of the membrane technologies is determined primarily by the development and investigation of suitable membranes. The main requirements for membranes are high selectivity, manufacturability, strength, good mass-dimensional characteristics, reliability, durability, and low cost [25,26,27]. The polymeric membranes are dominant in the market because they meet these requirements to a large extent. Selectivity and permeability both depend on the design, material, and structure of the membrane, as well as on the operating conditions—pressure and temperature [28,29,30,31].

The structure of polymeric membranes has important effects on the final separation of a mixture of two main components of biogas: methane CH_4_ and carbon dioxide CO_2_ through the membrane. To improve the permeation flux without reducing the selectivity, the thin-film composite membrane is a good alternative to dense membrane due to its ultrathin selective layer with lower mass transfer resistance and stronger mechanical support layer [32,33,34,35]. The composite membrane usually consists of several layers, such as the protective coating layer, selective layer, gutter layer, and support layer [36,37].

The selective layer is used to separate the gas mixture by allowing gas species of interest to pass through it while inhibiting the permeation of the other gas species. The most important characteristic of this layer is its selectivity (separation ability), which determines the efficiency of the membrane. Selectivity depends on the layer structure and material. The selective layer is made thin and dense in many different ways. The selective layer of the thin-film composite membrane was cross-linked by grafting cross-linker into the polyamide layer during the interfacial polymerization and by applying post thermal annealing to overcome the swelling issues and enhance the separation performance [38]. A mixed matrix selective layer comprising poly (ether-block-amide) (PEBA) and metal-organic frameworks (MOFs) was prepared on ceramic hollow fiber substrate by the dip-coating process to improve the pervaporation performance [39]. A more dense and cross-linked polyamide layer was fabricated by incorporating carboxylated TiO_2_ nanoparticles into the polyamide membrane matrix [40]. The thin-film nanocomposite membranes that consist of polyamide selective layer with incorporated multi-walled carbon nanotubes (MWNT) formed on polysulfone substrate was fabricated by interfacial polymerization method to improve the CO_2_ removal performance [41].

A protective layer with highly permeable property can be coated on the selective layer to seal the small defects in the selective layer and to protect the thin selective layer from chemical attack during its application or mechanical abrasion during the membrane module fabrication process [37,42].

A gutter layer [43,44,45,46] is often coated on the porous support prior to the selective layer to prevent the diluted polymer solution from penetrating into the porous structure resulting in the blockage of pores. Moreover, a gutter layer can modify or smooth the support layer to guarantee that a defect-free selective layer is coated on the support layer. The material of the gutter layer is usually highly permeable polymers. For instance, Liang et al. [47] applied cross-linked polydimethylsiloxane (PDMS) gutter layer between the selective layer and substrates to mitigate the adverse solvent effects during the dip coating and increase the adhesion of selective layer on the support layer.

The support layer is usually a porous layer made of low-cost materials with good mechanical properties. It does not perform separations but provides mechanical support for a selective layer. There are several different polymers used for the preparation of support layers, such as polysulfone (PSf) [48,49,50], polyacrylonitrile (PAN) [51,52,53], polyvinylidenefluoride (PVDF) [54], and polytetrafluoroethylene (PTFE) [55]. They are mechanically strong and chemically stable. The solution casting method is frequently used to prepare the support layer.

The main transport mechanisms for gas separation are Poiseuille flow, Knudsen diffusion, molecular sieving, capillary condensation, surface diffusion, solution-diffusion, and facilitated transport [56]. However, for the removal of CO_2_ from biogas by polymeric membranes with dense selective layer, solution-diffusion and facilitated transport mechanisms are the most favorable [57,58].

The solution-diffusion mechanism [59] is based on the solubility of specific gases within the membrane and their diffusion through the dense membrane matrix. In this mechanism, each of the gases is absorbed and dissolved on the upstream surface of the membrane and moved across the layer by diffusion. The diffusion flux of each gas is proportional to the difference of chemical potential between both sides of the selective layer. Moreover, the solubility and diffusion coefficient of gases in the layer, the characteristics of the polymer, and the physical-chemical interaction between gas species and polymers have a critical influence on the diffusion flux [60]. Due to the difference in the diffusion fluxes of gases, their separation can be very significant.

The facilitated transport mechanism is based on the chemical reaction between the gas of interest and the carrier loaded on the membrane. The reactive gas species are carried across the membrane easily, whereas the transport of non-reactive gases is inhibited. The driving force for gas transportation is the partial pressure difference across the membrane, however different reactive carriers can be used to increase the permeability and selectivity [14,58,61].

Polymer membrane materials must meet some common requirements, such as strength, durability, low manufacture costs, and energy consumption costs. However, the main requirement for membrane materials (the selective layer) is its high selectivity. Currently, materials such as cellulose acetate (CA) [62,63], polycarbonate (PC) [64], polyamide (PA) [65,66] and polysulfone (PS) [67] are frequently used for the membrane separation of CO_2_/CH_4_.

The numerous studies are aiming for further increase of the separation efficiency of membranes. The research is associated with the blending of additives (often other polymers) into the main material. An example of such a membrane of combined composition is polyphenylsulfone (PPSU). This is a membrane made from polysulfone blended with phenyl. Such membranes are applied to the separation of liquids [68,69,70]. There is only a few research on their application for gas separation [71,72], therefore, it is highly necessary to investigate the gas separation properties in further detail.

Polymeric membranes demonstrate the so-called trade-off relation between permeability and selectivity. Moreover, the gas separation performances of unmodified polymeric membranes cannot be improved above the Robeson upper bound due to the intrinsic properties of polymers [15]. Dispersion of nanoparticles into polymer matrix is an effective way to improve the gas separation performance of polymeric materials [73]. Therefore, silica nanoparticles synthesized in our laboratory were incorporated into the PPSU matrix to break the trade-off relation. The addition of nonsolvent into the polymer solution affected the phase inversion process, and consequently the membrane morphology, which additionally influenced the gas separation performance.

According to the literature data, flat sheet PPSU membranes are often prepared by solvent evaporation followed by the heat treatment for residual solvent removal [71,74]. The dense PPSU membranes prepared by this method possess low CO_2_ permeability due to the low intrinsic gas permeability of PPSU material [74]. To prepare PPSU based flat sheet membranes with high CO_2_ permeability and satisfying CO_2_/CH_4_ selectivity, a novel membrane fabrication method consisting of the addition of nanoparticles and/or nonsolvent into the polymer solution and the nonsolvent induced phase inversion process is developed in the present study. The addition of nanoparticles and/or nonsolvent to polymer solution can tune the morphology of membrane resulting in the improved CO_2_/CH_4_ selectivity.

In the present study, pristine PPSU membranes and mixed matrix PPSU membranes incorporated with silica nanoparticles were fabricated by nonsolvent induced phase inversion process. Silica nanoparticles as inorganic fillers were synthesized based on the Stöber method in the previous work [75]. The influence of additives in the polymer solution on the morphology, thermal properties, and gas separation of prepared membranes were investigated. The capabilities of PPSU based membranes for the CO_2_/CH_4_ separation were evaluated by experimentally studying the permeability and selectivity of CO_2_ and CH_4_ gases through various PPSU based membranes.

## 2. Materials, Experimental Equipment, and Methods

### 2.1. Materials

Polyphenylsulfone (Radel^®^ R-5000, PPSU, M_w_ = 50,000 g∙mol^−1^) was purchased from Solvay Advanced Polymer (Beveren, Belgium), *N*-methyl-2-pyrrolidinone (NMP, 99%) and glycerin were purchased from Acros Organics (Geel, Belgium), silica nanoparticles (SN) were synthesized based on the Stöber method described in detail elsewhere [75]. CO_2_ (99.99 mol.%) and CH_4_ (99.999 mol.%) gases were provided by AGA (Linde group, Vilnius, Lithuania).

### 2.2. Membrane Preparation

A Stovall Low Profile Roller (Stovall Life Science Inc., Greensboro, NC, USA) was used for dissolving PPSU pellets in solvents during the preparation of polymer solutions. For the fabrication of M1 membranes, 30 wt.% of polymer solution was prepared by dissolving PPSU pellets in NMP at ambient temperature. For the fabrication of M2 and M3 membranes, 27.5 wt.% of polymer solutions were prepared by dissolving the PPSU pellets in two solvent mixtures of NMP and glycerin at ambient temperature. 27.5 wt.% of polymer solutions containing silica nanoparticles (SN) content of 0.1 and 0.3 wt.% relative to the polymer concentration were prepared for the fabrication of M4 and M5 membranes. The silica nanoparticles were initially dispersed in NMP in an ultrasonic bath until a homogeneous dispersion was formed. The corresponding amount of PPSU resin was mixed with the silica dispersion until all the polymer resin was homogeneously dissolved in the solution. The present bubbles in all the polymer solutions were released by placing the polymer solution in a vacuum chamber with the bottle cap partially open. The composition of mixtures used for the preparation of the investigated membranes is summarized in Table 1.

A nonsolvent induced phase inversion method was applied to prepare PPSU based membranes. The bubble free polymer solution was firstly cast on a glass plate inside a controlled humidity (<40% RH) chamber using an automatically driven casting blade of 250 μm thickness (Convergence, Enschede, Netherlands). Then, the glass plate was immediately immersed in a coagulation bath containing demineralized water at 20 °C until the membranes peeled off from the glass plate. Finally, the membranes were rinsed with demineralized water and immersed in another demineralized water bath to remove traces of solvents.

### 2.3. Membrane Characterization

To explore the morphology and microstructures of membranes, images of the surface layer and the cross-section were taken by using a scanning electron microscope (Quantax 200 with an XFlash 4010 detector from Bruker AXS machine, Prague, Czech Republic). The scanning was performed at an accelerating voltage of 20 kV. Cross-section samples were prepared by fracturing the membranes in liquid nitrogen. Prior to SEM analysis, the samples were sputtered with a nanolayer of gold (5 nm Au layer thickness) to improve the conductivity of the samples.

The AFM measurements were performed on the Nanosurf Flex-Axiom microscope (Nanosurf, Liestal, Switzerland). The contact mode was selected to show the surface topography with the highest accuracy. ContAl-G probe (Nanosurf, Liestal, Switzerland) with spring constant 0.2 N/m dedicated for contact mode was used. Scan area of the sample was equal to 20 × 20 µm. In total, 256 scans in each direction were collected. The roughness parameters were determined using the Gwyddion 2.55 software. Each sample was analyzed at least three times and average values have been presented.

Thermal characterization of prepared polyphenylsulfone (PPSU) based membranes were analyzed using Simultaneous TGA-DTA Thermal Analysis TA Instruments type SDT 2960 (TA Instrument, Champaign, IL, USA). TGA measurements were performed in the temperature rangef 25–1000 °C under the ambient atmosphere of nitrogen and the heating rate of 10 °C/min.

Water (72.5 mN m^−1^), glycerol, (63.4 mN m^−1^), and α-bromonaphthalene (44.4 mN m^−1^) were used for the contact angle (CA) measurements. The selection of the testing liquids fulfilled the requirements of the Owens, Wendt, Rabel, and Kaelble method and the application of polar, bipolar, and nonpolar liquids. CA and topography measurements were done using Theta Flex Tensiometer (Biolin Scientific, Gothenburg, Sweden) at room temperature. Attension Theta (OneAttension Version 4.02) software was used for data acquisition and processing. The topography module works based on the fringe projection phase-shifting method. The advantage of the method is the possibility of a determination corrected value of the contact angle and surface free energy by the surface roughness. It gives the opportunity to distinguish the effect of surface chemistry and surface roughness on wettability. SFE was calculated using the geometric mean theory, proposed by Owens, Wendt, Rabel, and Kaelbe (OWRK) [76]. The detailed theoretical description of the OWRK method was presented elsewhere [77,78].

### 2.4. Experimental Set-Up for Gas Permeation Measurements

**Figure 2:**
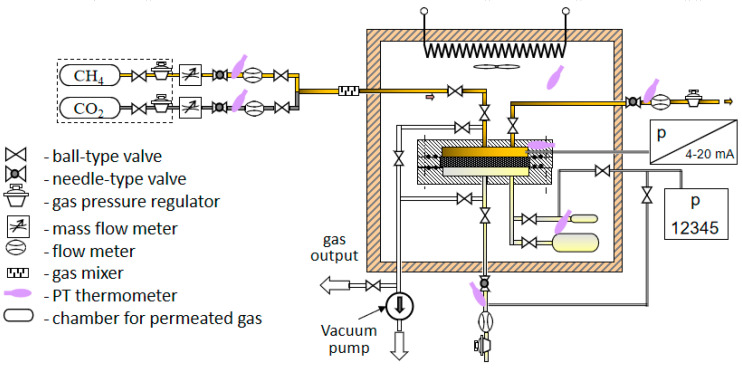
Experimental setup for membrane permeability experiments.

The experimental setup consists of the following main units: the membrane cell, thermostated chamber, system of storage and supply of permeating gases, gas flow measurement system, temperature measurement system, pressure measurement system, vacuum pump, and vacuum gauge. The design of the unit for membranes test is shown in Figure 2. Brassy housing of the cylindrical shape is dismountable in the horizontal plane to allow the change of membranes. The unit consists of the base with the tested membrane and membrane support with the cover. The massive housing from the metal of high thermal conductivity ensures uniformity and stability of gas and membrane temperature. The test membrane is cut from a polymer film; the diameter is the same as that of the support, i.e., 100 mm. The membrane fits tightly to the support surface from the gas inlet side due to the difference of pressures between the feed and permeate sides of the membrane. Gas permeates through the part of the membrane which contacts the perforated part of the support.

### 2.5. Gas Permeation Test

Pure CO_2_ and CH_4_ gases with purity 99.99 mol.% and 99.999 mol.%, respectively were used for the single gas permeation test, using the experimental apparatus shown in Figure 2. Pressure difference was created by the pressurized feed gas. The trans-membrane pressure was changed from 0.05 to 3 bar at a constant temperature of 25 °C. A membrane cell with an effective area of 38.47 cm^2^ was employed for gas permeation measurements. To ensure the accuracy of the experiments, the gas permeation measurement was repeated three times in the stabilized condition. The gas permeance was calculated by Equation (1):

(1) $$J=\frac{Q}{A\Delta p},$$

where *Q* is the flux of gas permeation rate (cm^3^ (STP)/s), Δ*p* is the pressure difference across the membrane (cmHg), *A* is the effective membrane area (cm^2^), and *J* is the gas permeance expressed in GUP (1 GPU = 10^−6^ cm^3^ (STP) cm^−2^ s^−1^ cmHg^−1^).

Permeability coefficient and the ideal selectivity are two important parameters employed to evaluate the membrane performance in the gas separation process [56]. The permeability coefficient (*P*) is the permeance normalized by membrane thickness (*l*), according to Equation (2):

J = P/l,(2)

The unit of permeation coefficient is Barrer, where 1 Barrer = 10^−10^ cm^3^ (STP) cm^−1^ s^−1^ cmHg^−1^.

The dimensionless ideal selectivity α_12_ is defined as the permeability coefficient or permeance ratio of two pure gases shown as follows (Equation (3)):

(3) $$\alpha_{12}=\frac{P_{1}}{P_{2}}=\frac{J_{1}}{J_{2}}.$$

## 3. Results and Discussion

### 3.1. Structure and Morphology of the Investigated PPSU Membranes

**Figure 3:**
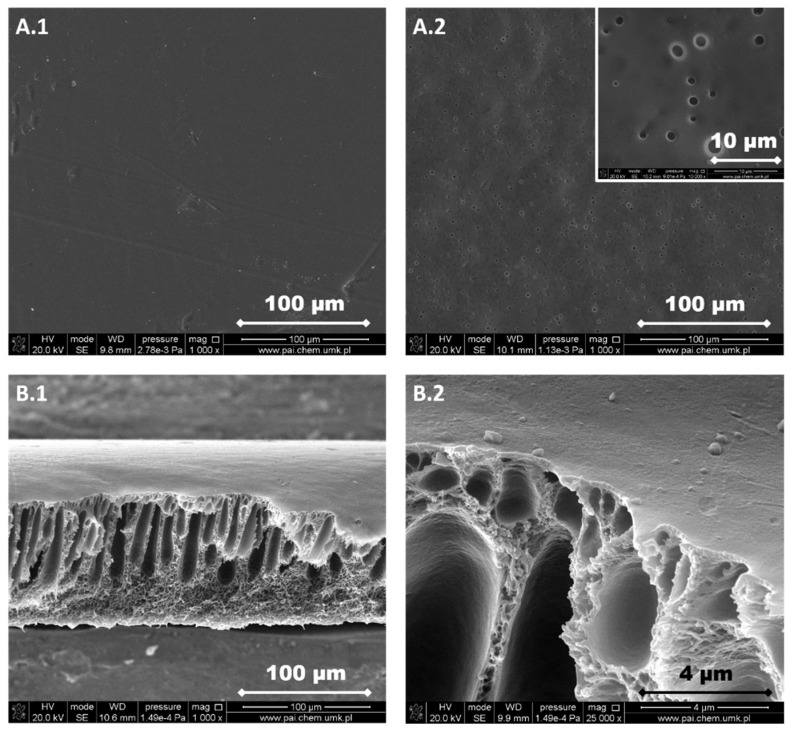
SEM images of M1 membrane. (**A.1**) Selective layer, (**A.2**) support layer, (**B.1**) a cross-section of the membrane, and (**B.2**) a cross-section of the membrane at higher magnification.

**Figure 4:**
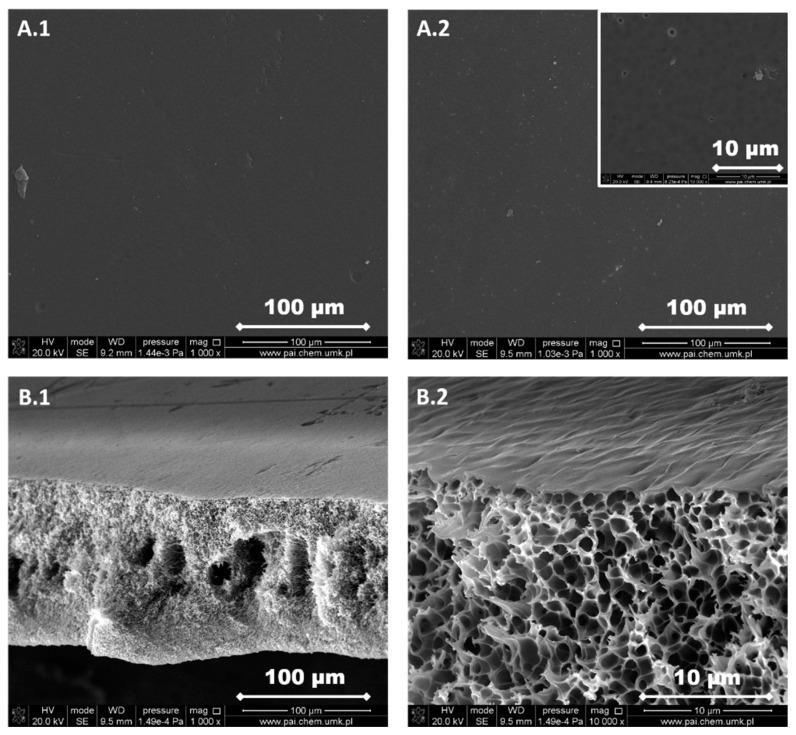
SEM images of M2 membrane. (**A.1**) Selective layer, (**A.2**) support layer, (**B.1**) a cross-section of the membrane, and (**B.2**) a cross-section of the membrane at higher magnification.

**Figure 5:**
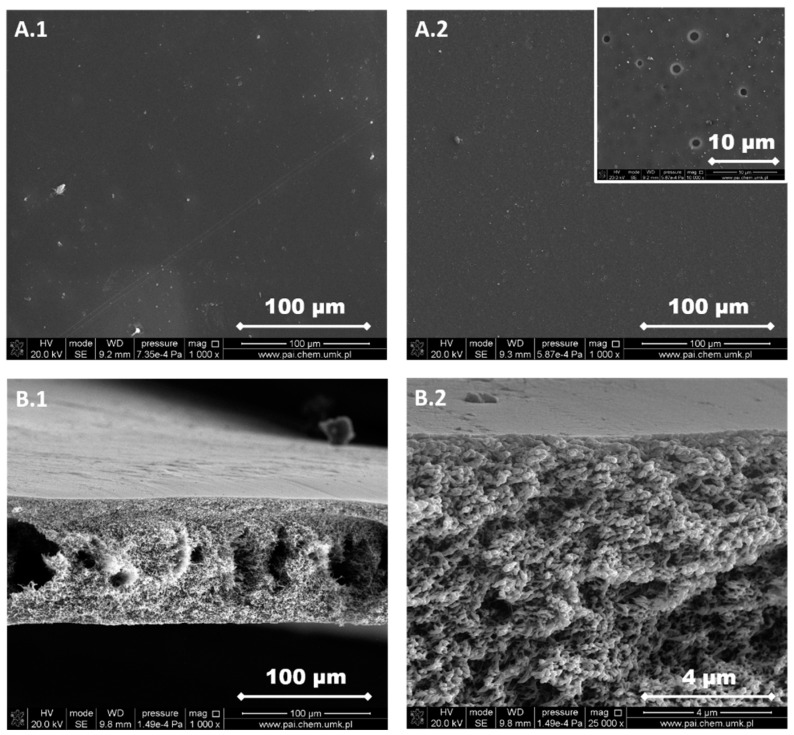
SEM images of M3 membrane. (**A.1**) Selective layer, (**A.2**) support layer, (**B.1**) a cross-section of the membrane, and (**B.2**) a cross-section of the membrane at higher magnification.

**Figure 6:**
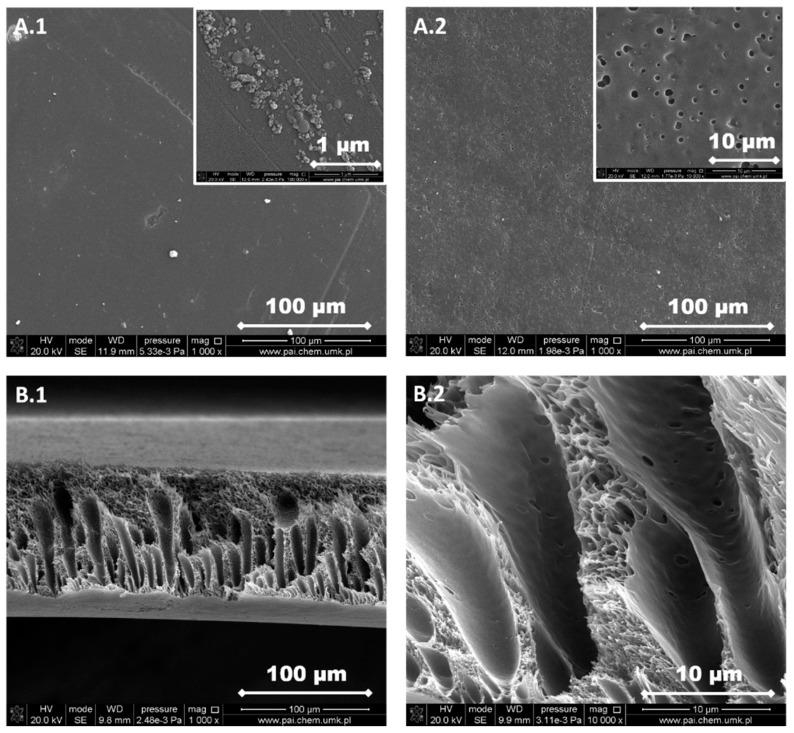
SEM images of M4 membrane (**A.1**) Selective layer, (**A.2**) support layer, (**B.1**) a cross-section of the membrane, and (**B.2**) a cross-section of the membrane at higher magnification.

**Figure 7:**
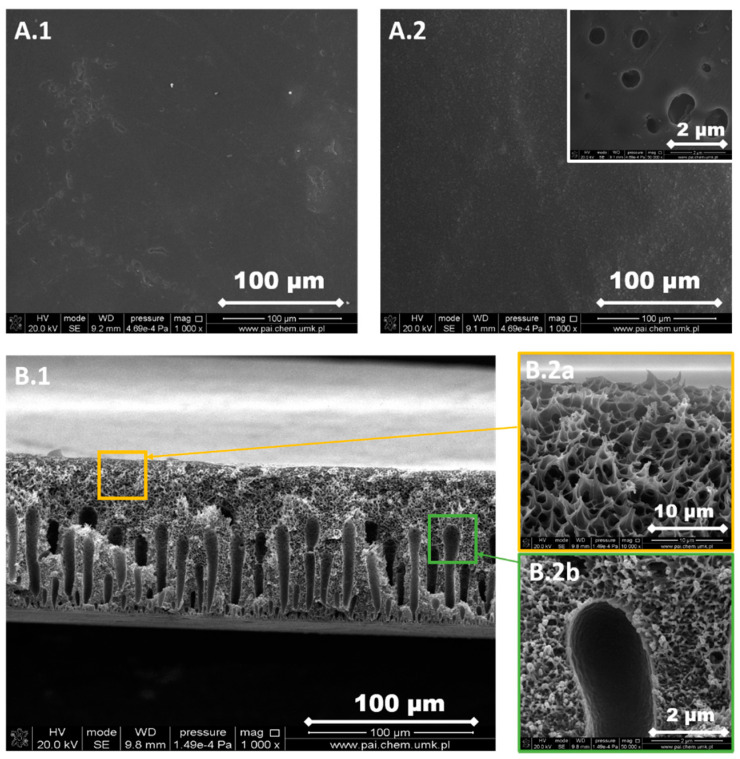
SEM images of M5 membrane. (**A.1**) Selective layer, (**A.2**) support layer, (**B.1**) a cross-section of the membrane, and (**B.2a,b**) a cross-section of the membrane at higher magnification.

The structure of each membrane was determined from the analysis of cross-section pictures (Figure 3, Figure 4, Figure 5, Figure 6 and Figure 7). The obtained pictures allowed us to understand the detailed morphology and structure of individual layers, as well as the structure of the whole membrane. The chosen morphological parameters, including the thicknesses of the selective and the support layer, estimated from SEM images, are summarized in Table 2.

M1 membrane possesses a thin dense selective layer (Figure 3). The surface of the support layer is porous. As can be seen from the cross-section of the membrane, this membrane contains a thin dense selective layer on the top surface of membrane followed by finger-like macrovoids in the support layer and a sponge-like structure at the bottom part of the support layer. The thickness of the selective layer is 0.33 μm.

The support layer does not provide the mass transport resistance due to its porous structure. M2 membrane possesses the dense outer surface of the selective layer and the porous inner surface of the support layer (Figure 4). The thickness of the selective layer is 3.8 μm. As the cross-section shows, the part between the outer dense selective layer and inner dense support layer has a sponge-like structure with micropores. The M3 membrane possesses a thin dense selective layer, with the thickness of 1.2 μm (Figure 5). The inner surface of the support layer is porous. Moreover, as the cross-section part shows, the support layer has a sponge-like structure with micropores. However, the micropores in the support layer of the M3 membrane are smaller than those in the support layer of M2 membrane. It was found that the addition of glycerin into the polymer solution allowed to avoid the formation of finger-like macrovoids and favors the formation of sponge-like porous structure in support layer during the nonsolvent induced phase inversion process. Increasing the content of glycerin in the polymer solution can decrease the size of micropores in the support layer. The structure of the support layer can be controlled by the addition of nonsolvent additives in the polymer solution. The effects of the addition of glycerin on the morphology and structure of PPSU membrane can be explained by the delayed liquid-liquid demixing process. It is well known that the instantaneous liquid-liquid demixing generally leads to the finger-like structure while the delayed liquid-liquid demixing is beneficial to the formation of the sponge-like structure. PPSU has good solubility in NMP as a solvent, while water is a strong nonsolvent. The addition of glycerin into the polymer solution is a useful way to control the phase inversion process and achieve desirable membrane morphology [79]. M4 membrane (Figure 6) also possesses a dense selective layer (0.43 μm). The major part of the support layer consists of the finger-like macrovoids followed by the dense selective layer. The minor part of the support layer displays a sponge-like structure at the bottom of the support layer. The M5 membrane (Figure 7) possesses a dense selective layer (1.7 μm) and a porous support layer. In contrast to M4 membrane, the support layer of M5 has a major part with sponge-like structure at the bottom of the support layer and a minor part with finger-like macrovoids follow the dense selective layer. Comparing morphologies presented in Figure 3, Figure 6, and Figure 7, it was found that the addition of 0.1 wt.% of silica nanoparticle into the polymer solution increased the number and length of finger-like macrovoids, however, when the content of silica nanoparticles is 0.3 wt.%, the formation of finger-like macrovoids was constrained to some extent. The membrane morphology change might be caused by the disruption of silica nanoparticles to the alignment of polymers.

All five types of PPSU based membranes possess a dense selective layer and the porous inner layer of support. The thickness of the selective layer is in the range of a few micrometers (Table 2). The support layers possess either a porous structure with a sponge-like structure and finger-like macrovoids, or only with a sponge-like structure. Both the addition of glycerin in the polymer solution and the incorporation of silica nanoparticles into the membrane affected the membrane morphology.

**Figure 8:**
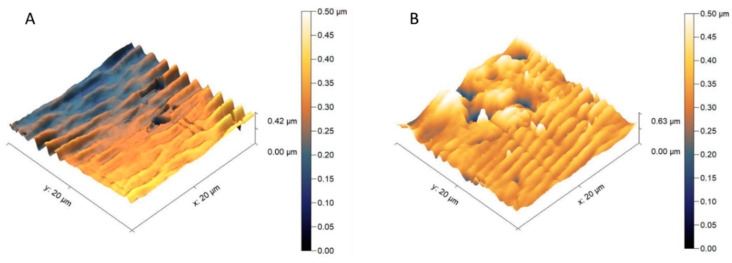
3D surface profile of pristine PPSU membrane M1 (**A**) and PPSU based mixed matrix membrane M4 incorporated with 0.1 wt.% of silica nanoparticles (**B**).

Roughness parameters for the investigated membranes were determined by the optical method (Figure S1) as well as by the implementation of atomic force microscopy (Figure 8).

The topography images of PPSU based membranes measured by the by Theta Flex Tensiometer equipped with 3D Topography module are presented in Supplementary Materials (Figure S1). As shown in the 2D and 3D topography images, no significant topography change was observed on the surface of the prepared PPSU based membranes. Therefore, to further discuss the surface morphology of PPSU based membranes, atomic force microscope (AFM) was implemented to measure the surface roughness (Figure 8). Pristine material possessed a lower roughness parameter in the comparison to the membrane filled with silica. Roughness parameters expressed by mean root square (Rq) were equal to 23.21 ± 1.20 nm for the pristine membrane and 58.42 ± 2.00 nm for the modified one, respectively. Due to the fact that the selected scanning area was high, the representative information about the material topography was presented. As shown in Figure 8, membrane M1 is smoother than membrane M4. Moreover, membrane M1 possesses regular wrinkle topography. In comparison with the pristine PPSU membrane (M1), the M4 membrane with incorporated silica nanoparticles, possesses the mountain-valley topography. The addition of silica nanoparticles into PPSU membrane changed the topography, and therefore the increased roughness of the membrane can be identified. Jullok et al. [75] investigated the topography of PPSU membranes and PPSU membranes with incorporated silica nanoparticles by using the non-contact mode of AFM. It was found that the silica nanoparticles incorporated PPSU membranes possess rougher surfaces (Rq is in the range 1.9–5.3 nm, depending on the content of silica nanoparticles) when compared with the pristine PPSU membranes (Rq = 1.7 nm). The roughness results from our investigation are different from the research of Jullok et al. [75], nevertheless, both studies found the same influence of the addition of silica nanoparticles into PPSU membranes on the surface roughness.

### 3.2. Thermal Properties

**Figure 9:**
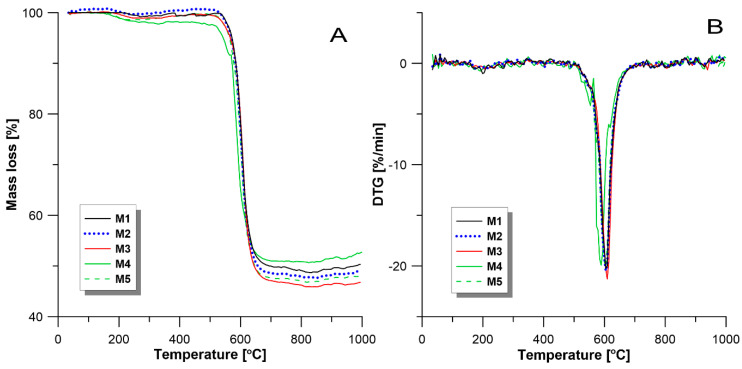
(**A**) represents the TGA curves of PPSU membranes and (**B**) represents the DTG curves of PPSU membranes.

The thermal properties of investigated membranes were characterized by TGA and DTG analysis. As shown in Figure 9, there are two weight loss stages of which a significant weight loss has occurred: the weight loss in the temperature range 550–650 °C representing final polymer decomposition; and the weight loss before 200 °C which might be due to the removal of resident solvent and the decomposition of branch chain. As shown in Table 3, the weight losses for membranes M1, M2, M3, M4, and M5 are 51.87%, 52.62%, 53.94%, 48.37%, and 52.49%, respectively. The temperature of decomposition of all membranes is around 605 °C, except for membrane M4 with the temperature of decomposition at 589.3 °C. T. Weng, et al. [74] observed that the PPSU homopolymer is thermally stable at temperature up to about 580 °C. These results indicate that all membranes possess good thermal resistance properties.

Dispersion of nano size fillers into polymer matrix and polymer-nanomaterial composite membranes is the alternative technology that has been tried to solve some of the issues of fouling, permeability, selectivity, and mechanical strength of membranes in the application of water treatment [80]. The prepared PPSU membranes possess high thermal stability with a decomposition temperature around 600 °C. PPSU membranes have been reported to possess desirable mechanical stability as evidenced by the finding that PPSU membranes barely swell in water at temperatures ranging from 20 to 150 °C [81]. Furthermore, the gas separation process is not operated at extremely high temperatures and pressures [82].

### 3.3. Contact Angle (CA) and Surface Free Energy (SFE)

**Figure 10:**
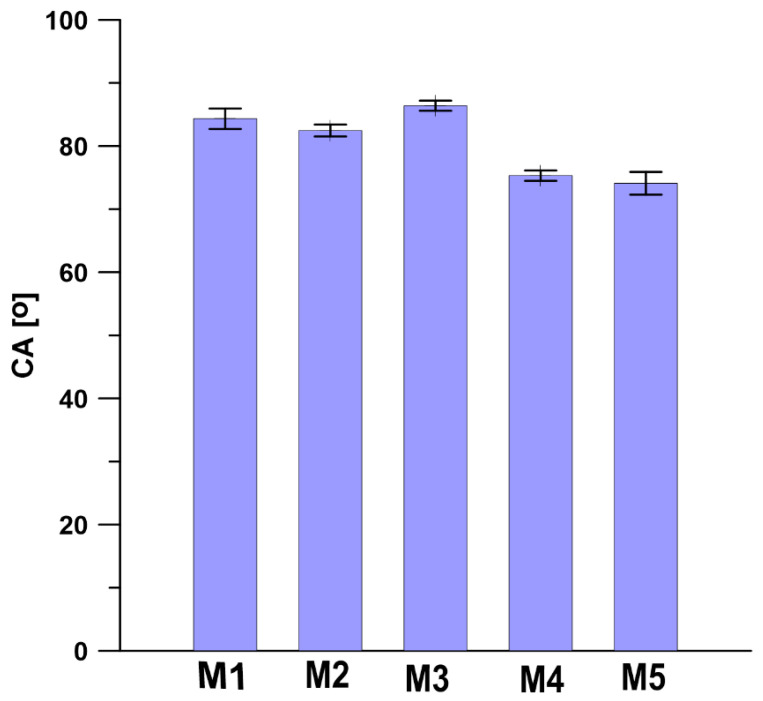
Water contact angle (CA) values of PPSU based membranes.

**Figure 11:**
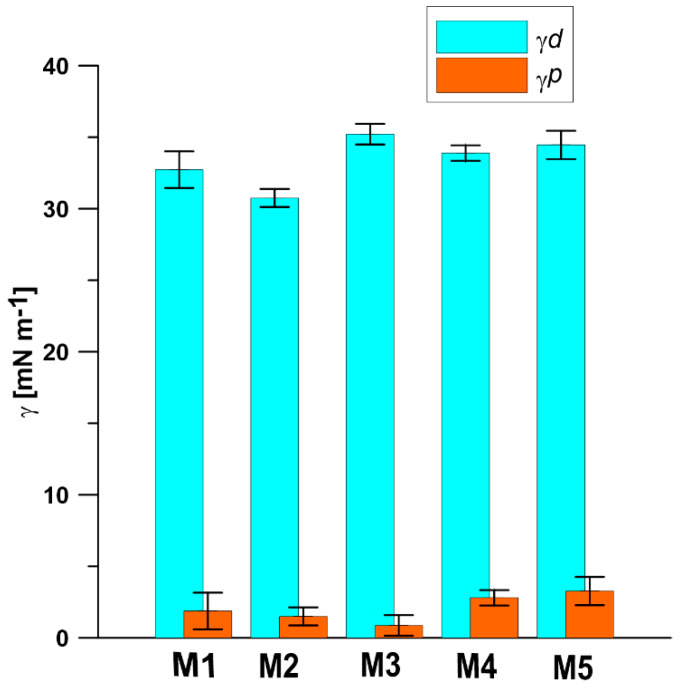
Surface free energy (γ) determined for PPSU based membranes surfaces. Superscripts d and p correspond to dispersive and polar components of surface free energy, respectively.

As shown in Figure 10, all PPSU based membranes possess the hydrophilic surfaces, as the water contact angle (CA) values determined on the surfaces of membranes are lower than 90°. The PPSU M3 membrane possesses the highest CA of 86.39°, while the membrane M5 incorporated with 0.3 wt.% silica nanoparticles possesses the lowest CA of 74.1°. Comparing the CA values of PPSU membranes prepared at different conditions (M1–M3, Table 1 and Figure 10) with the CA values of the silica nanoparticles incorporated into PPSU membranes (M4, M5, Table 1 and Figure 10) it can be seen that the presence of silica nanoparticles caused a slight decrease of CA (Figure 10) for the M4 and M5 membranes. The reduction of CA for membranes with incorporated silica nanoparticles results from the hydroxyl groups present on the silica nanoparticles in the PPSU membranes. This was also confirmed by an increase in the polar component of surface free energy of membranes with incorporated silica (Figure 11) [83]. Jullok et al. [76] investigated the influence of the incorporation of the pristine and modified silica nanoparticles into the PPSU based membranes on the performance of pervaporation dehydration of acetic acid aqueous solution. It was found that the incorporation of 0.5 wt.% pristine nanoparticles results in the reduction of water contact angle, indicating an increase in surface wetting. However, the incorporation of silica nanoparticles modified by 1,1,1,3,3,3-hexamethyldisilazane (HMDS) increases the water contact angle close to the hydrophobic region (90°) [75]. This was due to the replacement of hydrophilic (-OH) groups by the hydrophobic trimethyl (CH_3_)_3_ groups. Li et al. [84] found that the water contact angle reduced from 82.0° to 50.4° when the content of silica nanoparticles in polysulfone membranes increased from 0 to 20 wt.%. The water contact angle of membrane containing 5 wt.% of silica nanoparticles showed a water contact value of 76.2°, which is similar to the result obtained in this research (Figure 10). The reduction of the water contact angle indicates the presence of silica nanoparticles on the surface of the membrane. Ang et al. [84] added silica particles with different size into polyamide thin film composite nanofiltration membranes to evaluate the antifouling behavior of membranes. It was found that the addition of different size of silica particles has a similar effect on the hydrophilicity of the membrane. All the polyamide thin-film membranes with incorporated silica particles possess the water contact angle value of ca 20°, which was half of the water contact value of pristine polyamide thin-film membranes.

The surface free energy was calculated from the results of contact angle measurements of three various liquids, including polar and nonpolar liquids, on the surface of PPSU membranes by the OWRK/Fowkes method. As shown in Figure 11, the dispersive surface free-energy component has the highest contribution in total surface free energy for all PPSU based membranes. The polar component of surface free energy for PPSU membranes with incorporated silica nanoparticle is higher than that in pristine PPSU membrane, which is due to the hydrophilic (-OH) groups on silica nanoparticles. The addition of hydroxyl groups in PPSU membranes increases the polar part of surface free energy and the total surface free energy. The results of surface free energy are consistent with the water contact angle results. The higher polar part of surface free energy results in the lower water contact angle value.

### 3.4. Investigation of Gas Permeability and Separation Performance of Membranes

**Figure 12:**
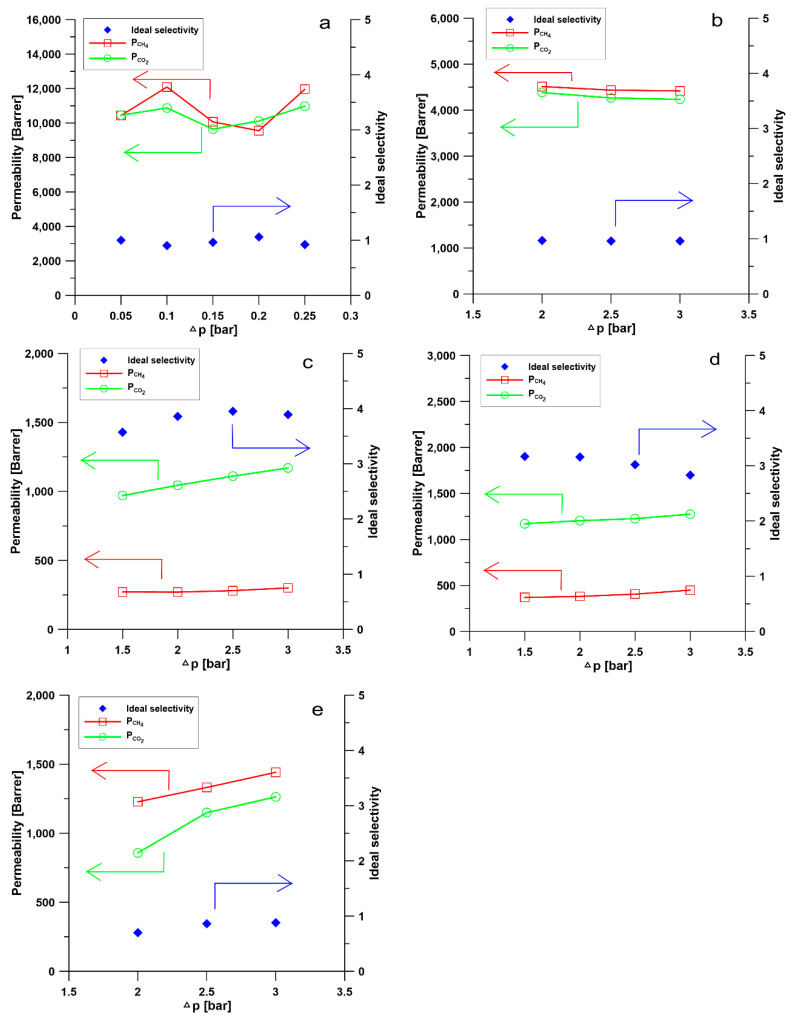
Permeability of CO_2_ and CH_4_ and CO_2_/CH_4_ selectivity through PPSU based membranes in which (**a**) represents data for M1, (**b**) represents data for M2, (**c**) represents data for M3, (**d**) represents data for M4, and (**e**) represents data for M5 membrane.

Permeation of pure gas CO_2_ and CH_4_ through different membranes at 25 °C and different pressure, from 0.5 to 3.0 bar, is presented in Figure 12. The comparison of gas separation performance of PPSU based membranes is shown in Table 4. The permeation of pure CO_2_ and CH_4_ and separation performance of membranes could be explained by the solution-diffusion mechanism. According to Equation (4), the permeability (*P*) of gases through polymeric membranes is defined as the product of solubility coefficient (*S*) and diffusion coefficient (*D*) [85].

*P* = *S D* (4)

The solubility coefficient reflects the affinity between gas molecules and polymeric membranes and it can be influenced by the condensability of penetrant gas. Gas molecules with higher condensability usually possess a larger solubility coefficient, such as CO_2_. The solubility coefficient describes the permeability from the aspect of thermodynamics, however, the diffusion coefficient reflects the mobility of gas molecules in the membranes matrix from the viewpoint of kinetics. The size of gas molecules and the fraction free volume (FFV) play crucial roles in determining the diffusion coefficient. The kinetic diameters of CO_2_ and CH_4_ are 0.33 and 0.39 nm, respectively. Moreover, carbon dioxide is more condensable than methane [67].

PPSU is a glassy polymer with a glass transition temperature of 220 °C [70]. The diffusion coefficient plays a predominant role when gas molecules penetrate through glassy polymer membranes. Naderi, et al. [86] found that the two additional aryl groups in PPSU result in a higher d-spacing (0.51 nm) and FFV, pore size, more local segmental motions and π flip motions of two aromatic rings. Consequently, PPSU membranes show low CO_2_/CH_4_ selectivity. Indeed, results in Figure 12a show that the M1 membrane possesses low CO_2_/CH_4_ selectivity around 1 and the permeabilities of CO_2_ and CH_4_ are similar (Table 4). This low selectivity and relatively high permeability can be attributed to the presence of aryl groups in the polymer structure, which results in higher FFV and larger pores. The permeabilities of CO_2_ and CH_4_ decreased with the increased amount of glycerin added into polymer solution (M2 and M3, Table 1 and Table 4, Figure 12b,c). The decrease in permeabilities of CO_2_ and CH_4_ can be explained by the increase in the thickness of selective layer and the change of membrane structure from finger-like to sponge-like (Table 2, Figure 3, Figure 4, Figure 5, Figure 6 and Figure 7). When 8 wt.% of glycerin was added into the polymer solution, the structure of membrane M2 possesses the sponge-like structure with larger micropores and the CO_2_/CH_4_ selectivity was even smaller than that of M1.

However, when the glycerin content increased to 10 wt.%, membrane M3 exhibited the highest CO_2_/CH_4_ selectivity of 3.86 but the lowest permeabilities of CO_2_ and CH_4_ among all the PPSU based membranes investigated. The addition of the appropriate amount of nonsolvent into polymer solution can influence the membrane morphology and structure, consequently, the gas permeability and selectivity. The decrease in permeabilities of CO_2_ and CH_4_ with the increase of pressure from 2 to 3 bar shown in Figure 12b, could be explained by the gas permeation through a dual-mode membrane described on the basis of the partial immobilization model [87]. It was also observed that the CO_2_ permeability increased with the increase in feed pressure while the CH_4_ permeability is practically constant in membrane M3 (Figure 12c), which is due to the CO_2_ plasticization effect [87]. The solubility of CO_2_ in a polar polymer is higher than CH_4_ because CO_2_ is more condensable and CO_2_ has a quadrupolar moment, which reinforces the interaction between CO_2_ and polar groups in the polymer [88]. The results presented in Figure 12d show, that the CO_2_ permeability is around 1200 Barrer and CO_2_/CH_4_ selectivity of 3.16. The incorporation of 0.1 wt.% of silica nanoparticles into the membrane resulted in the reduction of gas permeability but the increase in CO_2_/CH_4_ selectivity. However, when the content of silica nanoparticle increased to 0.3 wt.% the CO_2_ permeability decreased and the CO_2_/CH_4_ selectivity decreased (Table 4). The permeability of CO_2_ and CH_4_ increased with the increase of pressure difference. The incorporation of silica nanoparticles into PPSU membranes could affect the alignment of polymers, resulting in the change of polymer rigidity and size of free volume [89]. Consequently, the change of gas separation performance was observed. When the content of silica nanoparticles in the membrane is 0.3 wt.%, the selectivity decreased, which might result from the aggregation of nanoparticles [89].

The influence of the nonsolvent and silica nanoparticles on CO_2_/CH_4_ selectivity can be explained in the following way: the addition of nonsolvent into the polymer solution changed the morphology of PPSU membrane from finger-like to sponge-like with smaller micropores (Table 2, Figure 3, Figure 4, Figure 5, Figure 6 and Figure 7), which resulted in the increase of CO_2_/CH_4_ selectivity. The incorporation of silica nanoparticles into PPSU membranes could affect the packing of the polymer chains, inhibiting the chain mobility, and decreasing the size of free volume due to the stronger interactions between silica nanoparticles and polymer chains [88], which also resulted in the increase of CO_2_/CH_4_ selectivity. Another reason for the greater CO_2_/CH_4_ selectivity is the fact that the nonporous nano-sized particles (such as SiO_2_) have more affinity to CO_2_ in comparison with CH_4_ [90].

The stability of the separation performance is important for membranes used in gas separation process. All the prepared pristine and modified PPSU membranes exhibited high stability of CO2/CH4 selectivity during the whole gas separation tests. Basu et al. [91] have studied the gas separation stability of PSf membranes and blend membranes of PSf and Matrimid. It was found that all the PSf based membranes exhibited high stability of gas separation performance during the 14 h tests.

The performance of PPSU based membranes synthesized in other works is gathered in Table 5 and compared with the results obtained within this research. Weng et al. [76] prepared pristine PPSU membrane and the blend membrane of poly(bisphenol A-co-4-nitrophthalic anhydride-co-1,3-phenylenediamine (PBNPI) and polyphenylsulfone (PPSU). The blend membrane possessed higher CO_2_ permeability (34.1 Barrer) and CO_2_/CH_4_ selectivity (4.3) than the pristine PPSU membrane (Table 5). In comparison with their results, the membranes prepared within this study (M3 and M4) possess similar CO_2_/CH_4_ selectivity (3.86 and 3.16, respectively), however, the CO_2_ permeability was over 30 times higher (1044.0 and 1202.8 Barrer). Yong et al. [71] prepared polyphenylsulfone (PPSU) membrane for gas separation. In comparison with the performance of PPSU membranes in Yong et al. [71] work, membranes prepared in this work (M3 and M4) possess lower CO_2_/CH_4_ selectivity, however, more than 130 times higher CO_2_ permeability. The Pervaporation Separation Index (PSI) combining the permeability and selectivity can be used to estimate the separation performance of membranes in a given separation process. The higher the PSI value is, the more effective the membrane should be in the separation process [78]. As it is seen from Table 5, PPSU membranes (M3 and M4) prepared in this work possess significantly higher PSI value in comparison to the PSI value of PPSU membranes reported in the literature. Membranes with high PSI values are more suitable for their application in industrial biogas upgrading process. The combination of the addition of nanoparticles or nonsolvent into the polymer solution and the utilization of nonsolvent induced phase inversion process is a useful way to prepare PPSU based membranes with significantly high CO_2_ permeability and relatively satisfying CO_2_/CH_4_ selectivity for biogas upgrading.

## 4. Conclusions

The PPSU based membranes including pure PPSU membranes and mixed matrix membranes with incorporated silica nanoparticles were fabricated by solution casting and nonsolvent induced phase inversion methods. The membrane morphology and structure analysis from SEM images showed that the addition of glycerin as a nonsolvent in polymer solution can avoid the formation of finger-like macrovoids and change the porous structure of the support layer. It is crucial to adjust the amount of addition of non-solvent in the polymer solution to achieve the desirable membrane structure. The AFM images revealed that the addition of silica nanoparticles in PPSU membranes can increase the surface roughness. The thermal properties examined by TGA/DTG analysis showed that the PPSU membranes are highly thermally stable and the decomposition temperature is around 600 °C.

The gas permeation results showed that the addition of the appropriate amount of nonsolvent or nanoparticles into the polymer solution can influence the gas separation performance of prepared membranes. The membrane M3 prepared from 27.5 wt.% of PPSU polymer solution with 10 wt.% glycerin exhibited CO_2_ permeability of 1044.01 Barrer and the best CO_2_/CH_4_ selectivity of 3.86. The mixed matrix membrane M4 with 0.1 wt.% of silica nanoparticles showed a CO_2_ permeability of 1202.77 Barrer and the CO_2_/CH_4_ selectivity of 3.16.

## Figures and Tables

**Table 1:** Composition of the polyphenylsulfone (PPSU) based membranes.

Type of Membranes	Composition of Polymer Solution			
	PPSU [wt.%]	NMP [wt.%]	Glycerin [wt.%]	SN [wt.%]
M1	30.0	70.0	-	-
M2	27.5	64.5	8	-
M3	27.5	62.5	10	-
M4	27.5	72.4	-	0.1
M5	27.5	72.2	-	0.3

**Table 2:** The morphological characteristics of the prepared membrane.

Membranes	Membrane Thickness [μm]	Layer Function	Layer Thickness [μm]
M1	101 ± 3.9	Selective	0.33 ± 0.03
		Supporting	100 ± 3.8
M2	135 ± 1.0	Selective	3.8 ± 0.63
		Supporting	131 ± 0.86
M3	98 ± 1.0	Selective	1.2 ± 0.13
		Supporting	96 ± 0.89
M4	90 ± 4.5	Selective	0.43 ± 0.07
		Supporting	89 ± 4.6
M5	110 ± 0.35	Selective	1.7 ± 0.14
		Supporting	108 ± 0.30

**Table 3:** Thermal properties of PPSU based membranes.

Membranes	Mass Loss [%]	The Temperature of Decomposition [°C]	
M1	51.87	603.8
M2	52.62	605.3
M3	53.94	609.8
M4	48.37	589.3
M5	52.49	605.4

**Table 4:** Gas permeability and selectivity of PPSU based membranes measured at 2 bar and 25 °C, excepting M1 membrane measured at 0.2 bar at 25 °C.

Membranes	CH_4_ Permeability [Barrer]	CO_2_ Permeability [Barrer]	Selectivity CO_2_/CH_4_
M1	9553.51	10,118.37	1.06
M2	4514.3	4383.27	0.97
M3	270.42	1044.01	3.86
M4	381.06	1202.77	3.16
M5	1227.69	857.90	0.70

**Table 5:** The comparison of gas separation performances of PPSU based membranes.

Membranes	Permeability [Barrer]		Selectivity (α)	PSI* [Barrer]	Ref.
	CO_2_	CH_4_	CO_2_/CH_4_		
PPSU	8.0	0.3	25.00	192.0	[71]
PPSU	4.5	1.8	2.50	6.8	[74]
50PPSU/50PBNPI	34.1	8.0	4.30	112.5	
M1	10,118.4	9553.5	1.06	607.1	This work
M3	1044.0	270.4	3.86	2985.8	
M4	1202.8	381.1	3.16	2598.0	

* PSI = P (α – 1), where P is the gas permeability, and α is the selectivity [78].

## Supplementary Materials

The following are available online at https://www.mdpi.com/1996-1944/13/12/2847/s1, Figure S1: Topography images of PPSU based membranes measured by Theta Flex Tensiometer equipped with 3D Topography module. A1–A5 represent the 2D topography of membranes M1–M5, respectively and B1–B5 represent the 3D topography of membranes M1–M5, respectively.
