# Supplementary material for: Preparation and Characterization of Polyphenylsulfone (PPSU) Membranes for Biogas Upgrading
Source: Materials (Basel). 2020 Jun 25;13(12):2847. doi: 10.3390/ma13122847 (PMC7345145; doi:10.3390/ma13122847)
Supplement: Supplementary file 1 [file materials-13-02847-s001.pdf]

materials

MDPI

Supporting Materials

Preparation and Characterization of Polyphenylsulfone (PPSU) Membranes for Biogas Upgrading

Wojciech Kujawski [1,*], Guoqiang Li [1], Bart Van der Bruggen [2], Nerijus Pedišius [3], Jurij Tonkonogij [3], Andrius Tonkonogovas [3], Arūnas Stankevičius [3], Justas Šereika [3], Nora Jullok [2,4] and Joanna Kujawa [1,*]

1. Faculty of Chemistry, Nicolaus Copernicus University in Toruń, 7 Gagarina Street, 87-100 Toruń, Poland grantli@doktorant.umk.pl
2. Leuven KU, Department of Chemical Engineering, Process Engineering for Sustainable Systems, W. de Croylaan 46, BE-3001 Heverlee, Belgium; bart.vanderbruggen@kuleuven.be (B.V.d.B.); marie9581@yahoo.com (N.J.)
3. Laboratory of Heat-Equipment Research and Testing, Lithuanian Energy Institute, 3 Breslaujos Street, 44403 Kaunas, Lithuania; Nerijus.Pedisius@lei.lt (N.P.); Jurij.Tonkonogij@gmail.com (J.T.); Andrius.Tonkonogovas@lei.lt (A.T.); Arunas.Stankevicius@lei.lt (A.S.); Justas.Sereika@lei.lt (J.S.)
4. Centre of Excellence for Biomass Utilization, School of Bioprocess Engineering, Universiti Malaysia Perlis, Kompleks Pusat Pengajian Jejawi 3, Jejawi 02600, Perlis, Malaysia

* Correspondence: wkujawski@umk.pl (W.K.); joanna.kujawa@umk.pl (J.K.); Tel.: +48-56-611-4517 (W.K.); +48-56-611-4315 (J.K.)

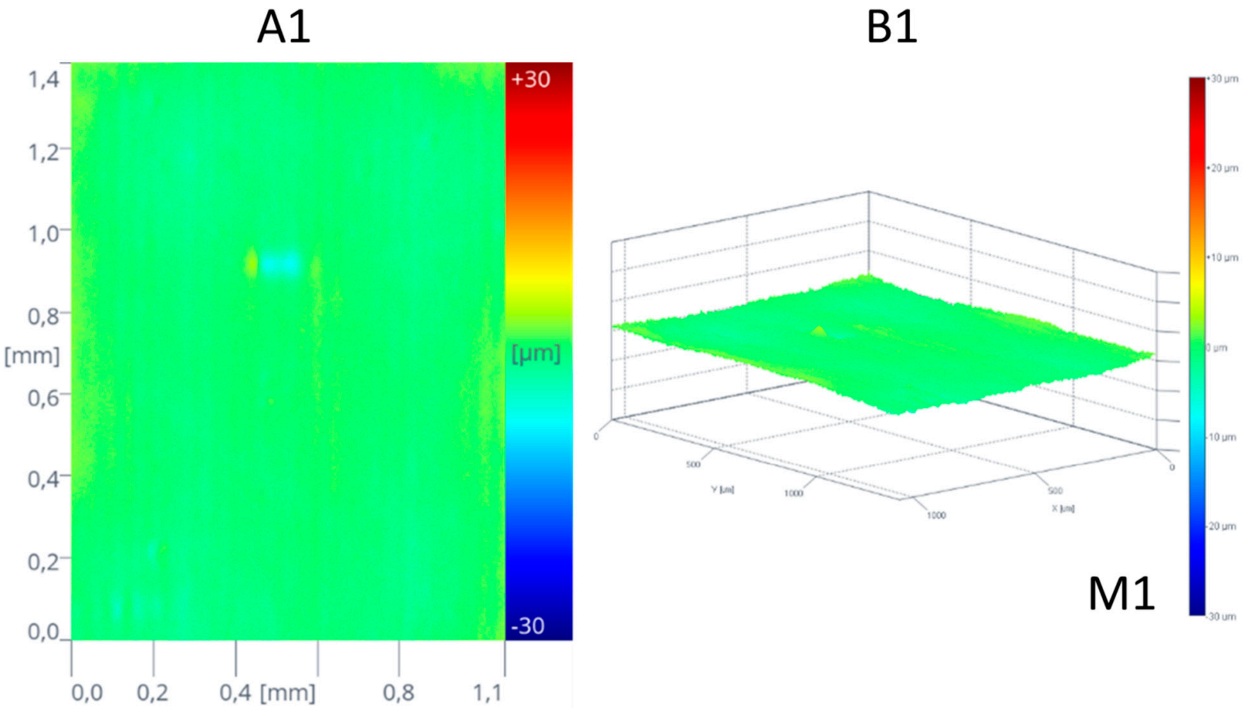

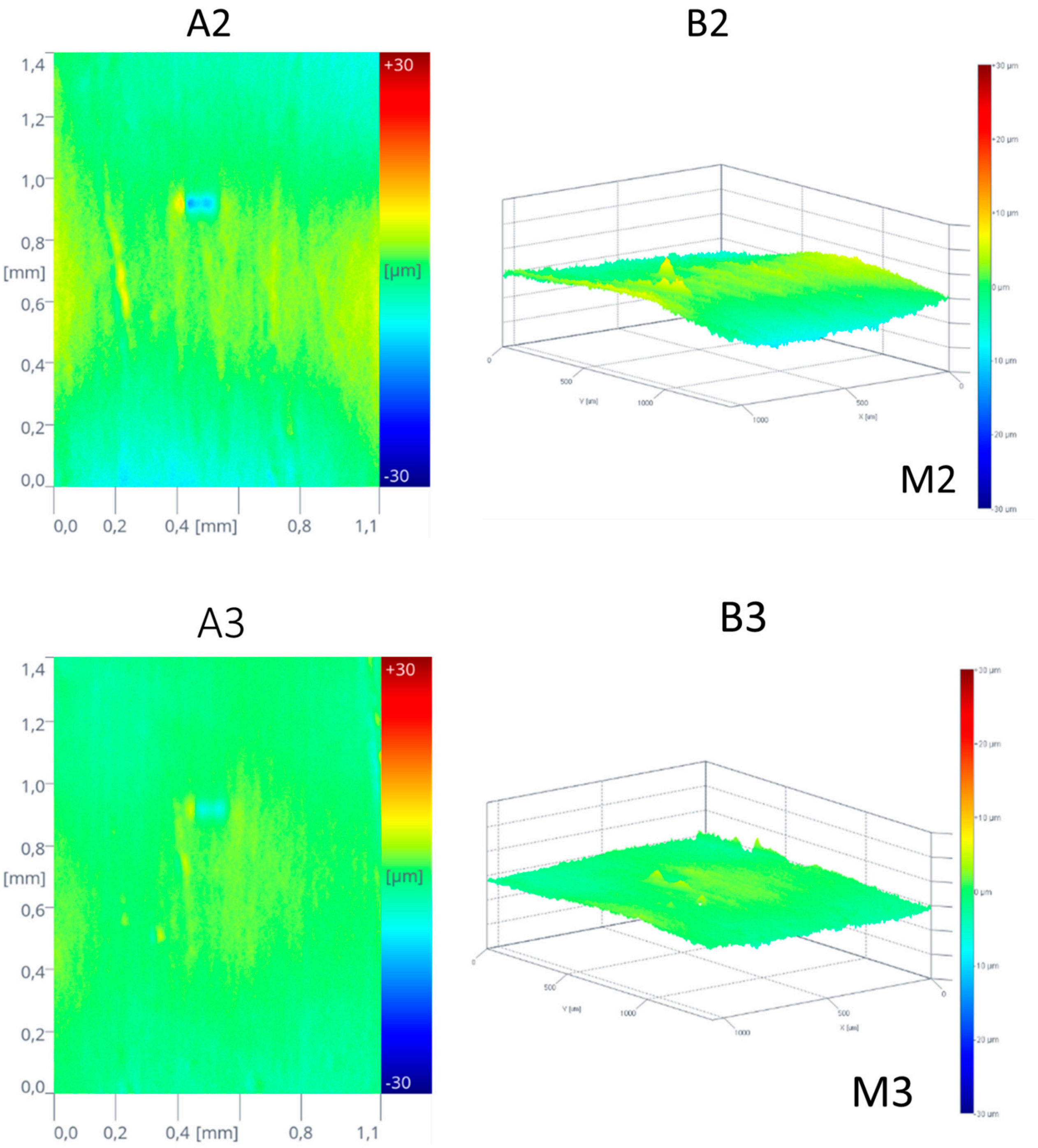
A2
B2
1,4
1,2
1,0
0,8
[mm]
0,6
0,4
0,2
0,0
0,0
0,2
0,4 [mm]
0,8
1,1
+30
[µm]
-30
M2
A3
B3
M3

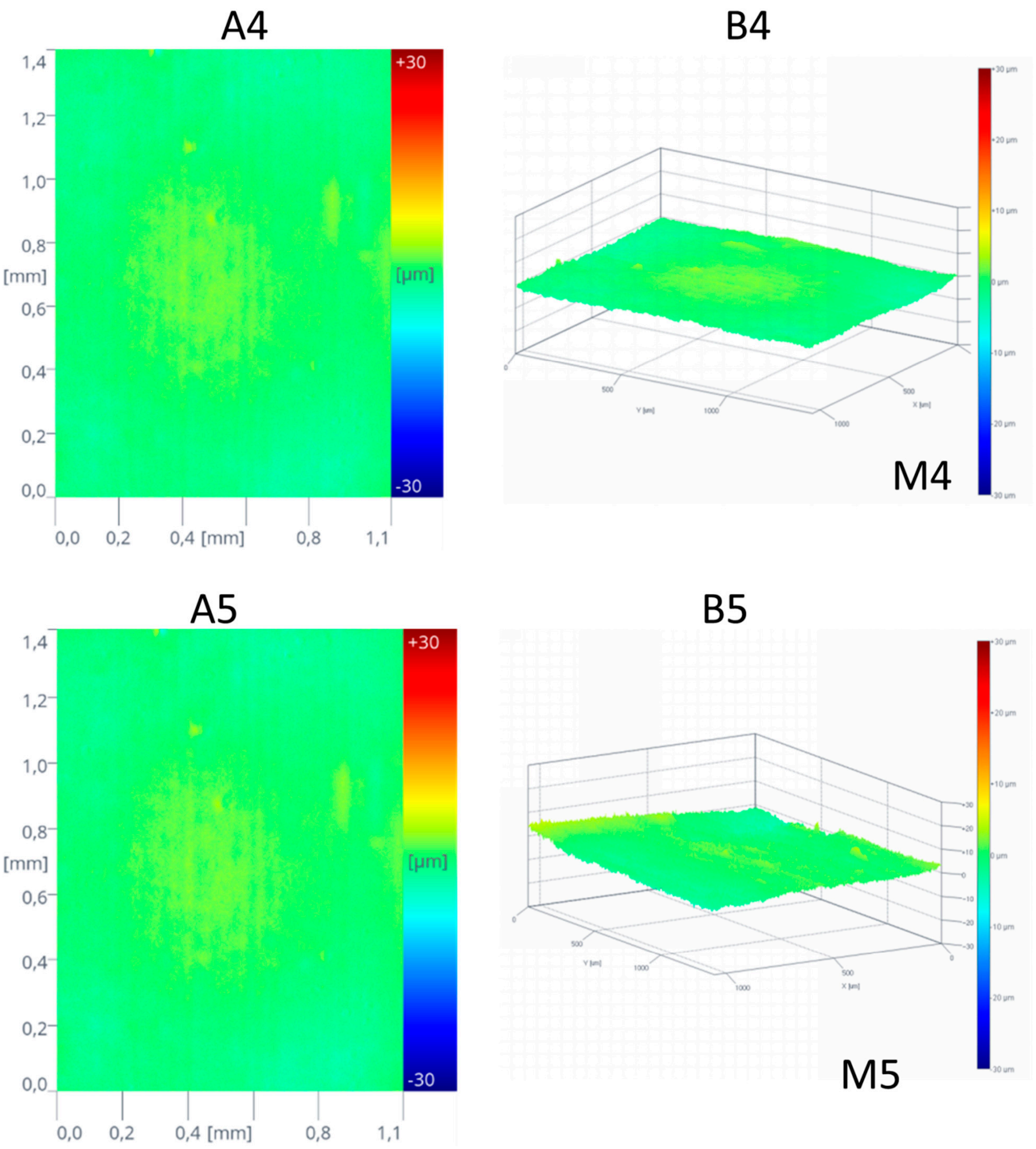

Figure S1. Topography images of PPSU based membranes measured by Theta Flex Tensiometer equipped with 3D Topography module. A1–A5 represent the 2D topography of membranes M1–M5, respectively and B1–B5 represent the 3D topography of membranes M1–M5, respectively.

© 2020 by the authors. Licensee MDPI, Basel, Switzerland. This article is an open access article distributed under the terms and conditions of the Creative Commons Attribution (CC BY) license (http://creativecommons.org/licenses/by/4.0/).
